# Lactate in the tumor microenvironment: A rising star for targeted tumor therapy

**DOI:** 10.3389/fnut.2023.1113739

**Published:** 2023-02-16

**Authors:** Zhangzuo Li, Qi Wang, Xufeng Huang, Mengting Yang, Shujing Zhou, Zhengrui Li, Zhengzou Fang, Yidan Tang, Qian Chen, Hanjin Hou, Li Li, Fei Fei, Qiaowei Wang, Yuqing Wu, Aihua Gong

**Affiliations:** ^1^Hematological Disease Institute of Jiangsu University, Affiliated Hospital of Jiangsu University, Jiangsu University, Zhenjiang, China; ^2^Department of Cell Biology, School of Medicine, Jiangsu University, Zhenjiang, China; ^3^Department of Gastroenterology, Affiliated Hospital of Jiangsu University, Jiangsu University, Zhenjiang, China; ^4^Faculty of Dentistry, University of Debrecen, Debrecen, Hungary; ^5^Faculty of Medicine, University of Debrecen, Debrecen, Hungary; ^6^School of Medicine, College of Stomatology, Shanghai Jiao Tong University, Shanghai, China; ^7^National Center for Stomatology and National Clinical Research Center for Oral Diseases, Shanghai, China; ^8^Shanghai Key Laboratory of Stomatology, Shanghai, China

**Keywords:** lactate, tumor microenvironment, metabolic, immunity, immune cells

## Abstract

Metabolic reprogramming is one of fourteen hallmarks of tumor cells, among which aerobic glycolysis, often known as the “Warburg effect,” is essential to the fast proliferation and aggressive metastasis of tumor cells. Lactate, on the other hand, as a ubiquitous molecule in the tumor microenvironment (TME), is generated primarily by tumor cells undergoing glycolysis. To prevent intracellular acidification, malignant cells often remove lactate along with H^+^, yet the acidification of TME is inevitable. Not only does the highly concentrated lactate within the TME serve as a substrate to supply energy to the malignant cells, but it also works as a signal to activate multiple pathways that enhance tumor metastasis and invasion, intratumoral angiogenesis, as well as immune escape. In this review, we aim to discuss the latest findings on lactate metabolism in tumor cells, particularly the capacity of extracellular lactate to influence cells in the tumor microenvironment. In addition, we examine current treatment techniques employing existing medications that target and interfere with lactate generation and transport in cancer therapy. New research shows that targeting lactate metabolism, lactate-regulated cells, and lactate action pathways are viable cancer therapy strategies.

## 1. Introduction

The cellular transformation includes uncontrolled cell proliferation, resistance to cell death, immune evasion, and evasion of growth inhibitory activity, ultimately leading to cancer formation (1, 2). Furthermore, it has been observed that, as part of the tumor survival machinery, tumor cells adapt to different survival challenges by altering their metabolism, a feature now considered a hallmark of cancer (1–3). Most normal tissues obtain energy through aerobic respiration in the presence of oxygen, while energy is provided only in the absence of oxygen through glycolysis. However, in tumor tissues, tumor cells select a low adenosine triphosphate (ATP)-generating mode of glycolysis to provide energy for their rapid growth and proliferation, even under adequate oxygen conditions, a specific phenomenon now known as the Warburg effect (4, 5), also known as glycolysis. The reasons why tumor cells choose this seemingly uneconomical method of metabolism include the fact that glycolysis allows tumor cells to better adapt to fluctuations in oxygen partial pressure. The intermediates of glycolysis can be used by tumor cells to synthesize macromolecules such as proteins, nucleic acids, and lipids needed for cell construction, thus maintaining the growth and proliferation of the tumor cells themselves, although glycolysis produces less ATP than aerobic respiration, it produces ATP at a higher rate to meet the energy demands of rapid tumor growth, and using glycolysis as the primary mode of energy supply reduces the mitochondrial of the electron transport chain (6), the production of anaerobic glucose glycolysis reduces the production of free radicals (ROS) and thus reduces the toxicity to tumor cells (6). Cancer cells use large amounts of glucose as their energy source, leading to the accumulation of extracellular lactate, which can alter the metabolic patterns of multiple cells, including immune cells, within the tumor microenvironment (TME) (7). In the early stages of tumor growth, immune cells recruited and activated by tumor cells can form a tumor-suppressive inflammatory microenvironment that hinders tumor progression. However, as tumor cells continue to proliferate and continue the immune activation response, the TME undergoes dynamic changes: immune effector cells become depleted or remodeled thus failing to function properly. An important cause of these changes is the high concentration of lactate produced by the Warburg effect, which affects the differentiation, metabolism, and function of tumor-infiltrating immune cells through several pathways (8). For many years lactate has been considered a waste metabolite, but it is now clear that lactate plays an irreplaceable role in promoting tumor cell survival, oncogene signaling (9), inflammation, metastasis (10), tumor resistance (11), immunosuppression (12, 13), and many other oncogenic processes. In this review, we briefly discuss the role of lactate in tumor progression, particularly its role in the metabolic microenvironment, immune microenvironment, and metastasis. We conclude that studies of targeted lactate therapeutic strategies and transport and combination therapy with other agents offer a new way of thinking to attack cancer and discuss the potential for clinical translation of lactate therapy.

## 2. The role of lactate in promoting tumor progression

### 2.1. The role of lactate in tumor cell metabolism

**Figure 1:**
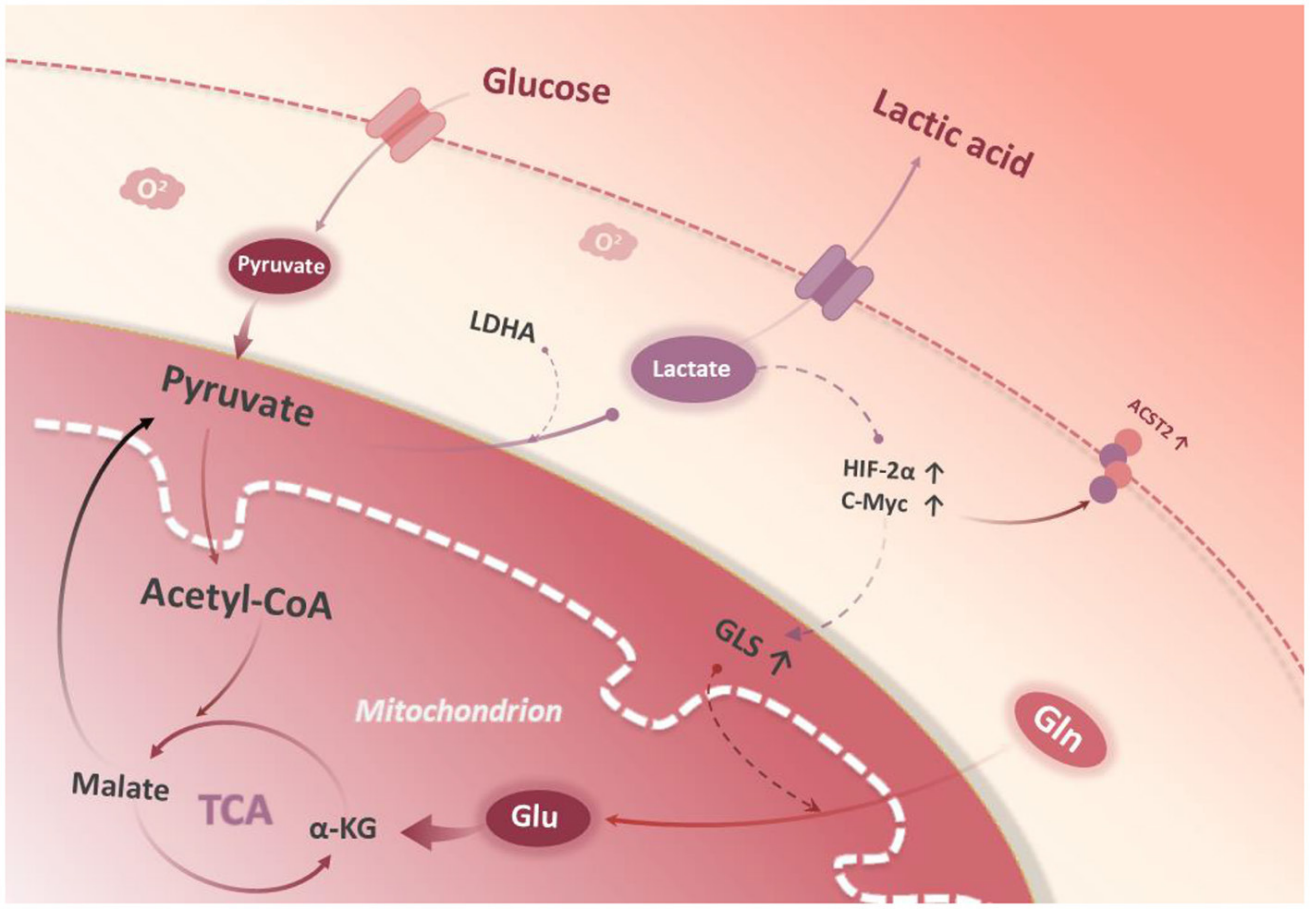
Glycolysis and glutamine metabolism. Glucose is catabolized to pyruvate in the cytoplasm. Conversion of lactate from pyruvate stabilizes HIF-2a, which then stabilizes c-Myc protein expression in the nucleus. c-Myc promotes glutamine transporter protein 2 (ASCT2) expression. Glutaminase 1 (GLS1) catalyzes the breakdown of glutamine to glutamate in the mitochondria. In the mitochondrial matrix, glutamate is converted to a-KG and enters the tricarboxylic acid cycle.

It has been found that lactate can stabilize HIF-2α and activate c-Myc, which results in an up-regulation in the expression of glutamine transporter (ASCT2) and GLS1, and an enhancement in glutamine uptake as well as catabolism (14). Damiani et al. found that tumor cells can maximize the use of α-ketoglutaric acid (α-KG), a catabolite of glutamine, into the TCA cycle, which is converted to pyruvate by malase and finally to lactate by LDHA (15) (Figure 1). The above preliminary clues suggest that glycolysis and glutamine catabolism pathways may be interdependent and jointly maintain the interstitial phenotype of tumor cells, but it is not clear whether there is a regulatory relationship between key enzymes LDHA and GLS1.

As a “mitochondrial vent”, PYCR dissipates electron aggregation by oxidizing NADH to NAD^+^, allowing the TCA cycle to proceed independently of oxygen consumption (16). This is also necessary for the oxidation of NADH produced by the glycolysis pathway (17). In addition, early reports found that lactate could directly inhibit the activity of proline oxidase (PRODH/POX) (18), and indirectly negatively regulates the expression of PRODH/POX, while reducing the degradation of Pro (19–21). Lactate can activate c-Myc to up-regulate the expression of PYCR, thus up-regulating the expression of proline. The above results suggest that lactate plays a positive regulatory role in the process of the Pro synthesis pathway.

### 2.2. Effect of lactate on tumor immunity

#### 2.2.1. Lactate and innate immune cells in the TME

Through the activity of its stromal cells, TME is in a state of continuous modification with the progress of the tumor. As an important site for tumor cells to survive, TME has a complex composition, including immune cells, cancer associated fibroblasts (CAFs), vascular endothelial cells, and other types of cells, an extracellular matrix, and a large number of active molecules.

**Figure 2:**
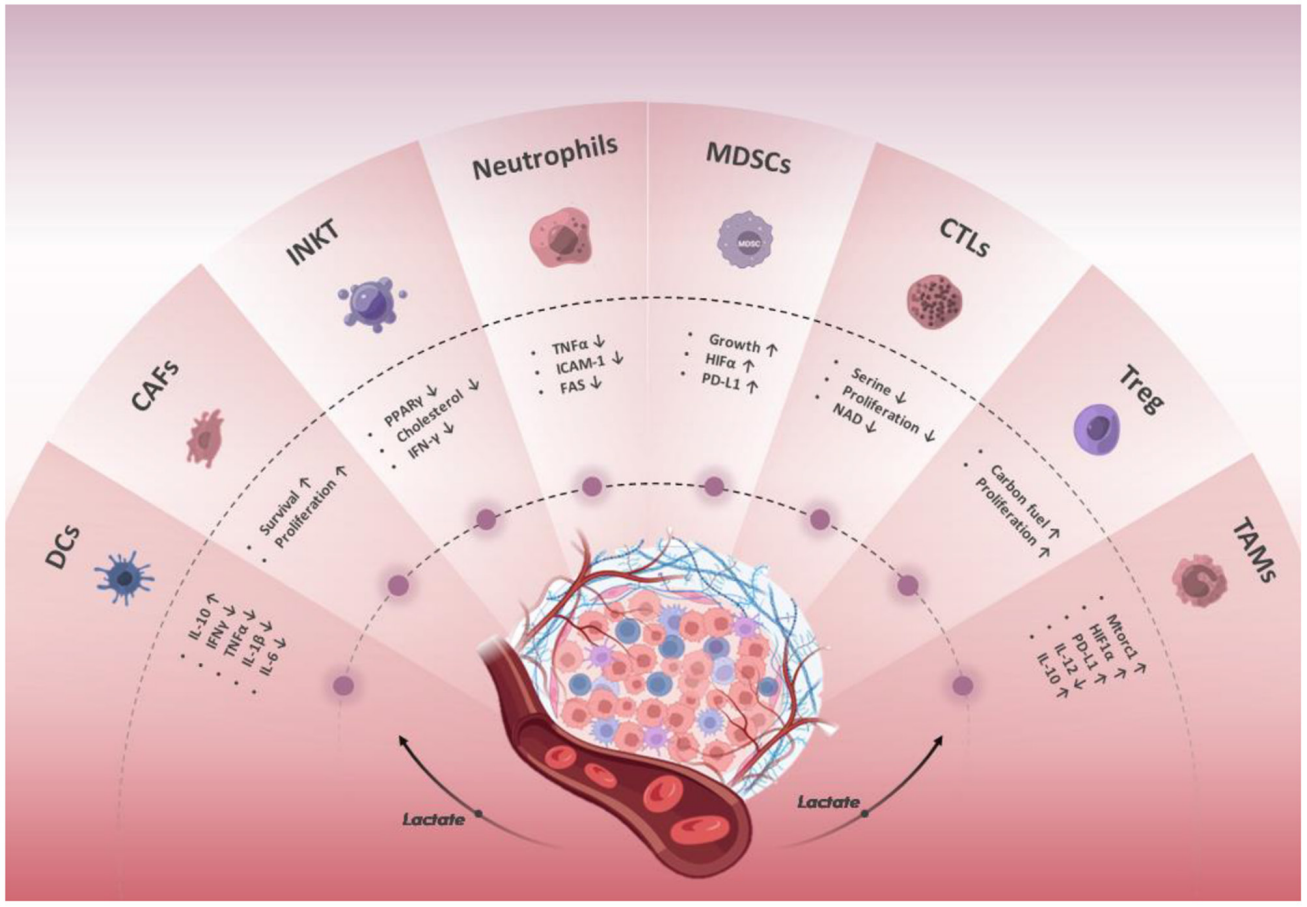
Effect of lactate in the regulation of the immune response. The lactate in the TME affects the differentiation, metabolism, and function of innate and adaptive tumor-infiltrating immune cells through multiple pathways and inhibits anti-tumor immune responses. The lactate secreted by tumor and stromal cells acidifies the TME and promotes tumor cell proliferation and metastasis.

On the one hand, innate immune cells present in TME, including macrophages, neutrophils, dendritic cells, natural lymphoid cells, invariant natural killer cells, and myeloid-derived suppressor cells, as well as adaptive immune cells including T and B cells, are responsible for detecting and eliminating cancer cells (22, 23). On the other hand, tumor cells can recruit immunosuppressive cell populations into TME by secreting anti-inflammatory cytokines, where they directly suppress immune responses (24) (Figure 2).

Invariant natural killer T (iNKT) cells are at the forefront of the anti-tumor immune response. They can not only kill target cells directly through antigen recognition but also enhance anti-tumor immune responses by inhibiting tumor-associated macrophages (TAMs) and promoting the activation of NK and Cytotoxic T lymphocytes (CTLs) (25–28). PPARγ and BTB–zinc finger transcriptional regulator PLZF synergically promote lipid biosynthesis of iNKT cells after activation through enhancing transcription of SERBP1. In the tumor microenvironment with high concentrations of lactate, the expression of PPARγ in intratumoral iNKT cells will be inhibited, thus consequently reducing their cholesterol synthesis. And cholesterol is necessary for iNKT cells to produce the optimal IFN- γ (29).

Tumor-associated macrophages are the most numerous subsets in the immune microenvironment, accounting for more than 50% of the immune cells in the tumor microenvironment (30). There are two main phenotypes of TAM: M1-TAM (tumor suppressor) has the function of antigen presentation and can activate immune factors, which is beneficial to anti-tumor immune response. M2-TAM (tumor-promoting type) can inhibit the inflammatory reaction, shield tumor immune surveillance, and promote tumor growth and metastasis (31). Lactate can activate the MCT-HIF1α pathway and promote macrophages to M2 polarization, which further complements the regulatory mechanism of macrophage polarization (32). The expression of IL-12 in M2-TAM decreased and the expression of IL-10 increased, which could promote the occurrence and growth of tumors. Experiments have shown that TAMs can regulate cancer progression through a variety of mechanisms (33). Lactate can also inhibit the degradation of HIF2α by activating mTORC1 in macrophages, thus promoting tumor development (34). Lactate inhibits the activation of YAP and nuclear factor-kB through a GPR81-mediated signal and reduces the production of pro-inflammatory cytokines in macrophages, thus inhibiting the pro-inflammatory response of macrophages to LPS stimulation (35). Regulation of lactate levels can promote the transformation of macrophages from M1 to M2 and upregulate PD-L1 to help tumor immune escape (36). In addition, studies have shown that reducing the level of lactate in tumors can inhibit the polarization of macrophages to M2, thus inhibiting the secretion of CCL17 and finally inhibiting the invasion of pituitary adenomas (37).

Myeloid-derived suppressor cells (MDSCs) represent a group of expanded, heterogeneous immature myeloid cells that can be subdivided into monocytic MDSCs (M-MDSCs) and granulocyte MDSCs (G-MDSCs). These two MDSC subsets are thought to have a significant ability to block innate and adaptive immunity (38). Several studies have shown that lactate in the acidic tumor metabolic microenvironment induces increased HIFα in MDSCs, leading to increased expression of programmed death ligand 1 (PD-L1), which regulates the development of myeloid cells (39, 40). Notch-RBP-J signaling plays a key role in determining cell fate and plasticity, and it is highly conserved (41). Recent studies have shown that myeloid-specific activation of Notch/RBP-J signaling could inhibit the transcription of the lactate transporter MCT2 through its downstream molecule Hes1, leading to a decrease in intracellular lactate levels and inhibition of granulocyte MDSC (G-MDSC) differentiation. Combining Notch activation and MCT2 inhibition in myeloid cells represses tumor growth (37).

DCs are the most functional specialized antigen-presenting cells found *in vivo* and they play a crucial role in initiating specific antitumor T-cell responses (42). One of the main functions of DCs is to activate the immune response by processing and presenting antigens through the MHC-II and MHC-I (43). The accumulation of lactate in tumors prevents the differentiation of DCs and makes the cells tolerant (44), limits the ability of DCs to recognize and present antigens (45), inactivates cytokines released by DCs, and promotes the production of an important immunosuppressive cytokine, IL-10, by DCs and inhibits the secretion of the pro-inflammatory factor IL-12 (46, 47). The effect of lactate on DC cells was also reversible when lactate was neutralized with NaOH to pH 7.4, showing that the inhibitory effect of lactate on DC activation disappeared (48).

Neutrophils are derived from bone marrow hematopoietic stem cells and have unique morphology because of their lobular nuclei, and can be determined by the phenotypes expressed on the cell surface. Neutrophils account for a significant proportion of the primary tumor immune cell infiltration (49). Recent studies have shown that neutrophils have both anti-tumor and tumor-promoting effects in cancers (50). N1 neutrophils are thought to have the potential to kill tumor cells due to the activation of immune factors such as TNF-α, ICAM-1, and FAS, resulting in elevated levels of immune factors and direct antibody-dependent cytotoxicity (51). N1 neutrophils can also exert anti-tumor effects indirectly through the regulation of T-cell function (52). It has been shown that N1 neutrophils exert antitumor effects through the release of neutrophil elastase (53). Neutrophils can undergo phenotypic and functional remodeling in response to lactic acid, which is the effect of N2. N2 neutrophils release a variety of pro-tumor factors and participate in promoting tumorigenesis and progression through multiple mechanisms. N2 neutrophils can release a variety of proteases that promote malignant proliferation and metastasis of tumor cells. matrix metalloproteinases (MMPs) mainly regulate tumor angiogenesis and metastasis. Blocking or reducing the production of these proteases is expected to achieve the purpose of inhibiting tumor progression (54).

#### 2.2.2. Lactate and T cells in the TME

T cells have long been thought to be effective against tumor cells and form lasting immunity, and their specificity to antigens expressed by tumors is crucial (55), but other inherent characteristics of T cells, such as persistence, longevity, and function, also play an important role in determining the effectiveness of immunotherapy (56). Tumor-derived lactate reduces CTL recruitment in TME and further inhibits the function of infiltrating CTL in TME by impairing its chemotactic and respiratory activities (57). Quinn et al. have reported that the inhibitory effect of lactate on the proliferation of effector T cells does not depend on acidity, which is achieved by the transition from NAD^+^ to NADH (lactate-induced reductive stress). This impairs glycolysis and the production of glucose-derived serine, which is necessary for effector T-cell proliferation (58). Tumor cells often exhibit uncontrolled metabolic processes, leading to a tumor microenvironment of metabolite depletion, hypoxia, and acidity, which makes it difficult for effector T cells to exert their lethal function. Recent studies have also found that lactate can promote the stem cell-like characteristics of CD8^+^T cells, thus playing an anti-tumor immune role in cancer treatment. This shows that lactate has two sides in T cell anti-tumor immunity (59). Regulatory T Cells (Tregs) also play an important role in immune homeostasis, and unlike other immune cells, Tregs have increased activity and recruitment in acidic TME, becoming a major barrier to anticancer immunity (60). Lactate can be used as a carbon fuel source for Treg to maintain its high inhibition ability. Inhibition of Monocarboxylate transporter 1 (MCT1), direct targeting of lactate metabolism, or inhibition of tumor acidity may break the metabolic symbiosis between tumor cells and Treg cells, thus reducing the Treg barrier of tumor immunity and enhancing the killing function of effector T cells (61).

#### 2.2.3. Lactate and CAFs in the TME

Cancer-associated fibroblasts (CAFs) are the predominant stromal cells in TME (62, 63). A vast number of studies have found that CAFs play a multifaceted function in tumor growth (63–66). They could not only provide ATP to adjacent cancer cells, but also regulate the tumor cells and tumor microenvironment by secreting various growth factors, cytokines, and chemokines [48], and prevent the deep infiltration of drugs and immune cells into tumor tissue by shaping the tumor extracellular matrix and forming a drug or therapeutic immune cell permeation barrier, thereby diminishing the therapeutic effects of the anticancer drugs (67, 68). In terms of offering energetic sources for tumor cells, CAFs emit a substantial quantity of lactate through glycolysis to the tumor cells which in turn use it as a fuel to support their physiological activities (69). A recent study demonstrated evidence in which the tumor-secreted lactate downregulates p62 in stromal fibroblasts, which essentially induces the CAF phenotype (70, 71).

## 3. Mechanism of lactate inhibition of antitumor response

### 3.1. Gene regulation by histone lactylation in tumor cells

Lactate modification (lactylation) is a histone post-translational modification reported by Zhang et al. for the first time, which plays a role in gene transcriptional regulation (72). Follow-up studies have further confirmed that protein lactylation is an important way for lactate to exert its function and participate in cellular life activities such as glycolysis-related cell function, macrophage polarization, nervous system regulation, and so on (73–75). The discovery of histone lactylation has pointed out a new direction for the research on the participation of tumor cell metabolites in the tumor, immunity, and other fields. The researchers found that lactylation of histone lysine residue, as an epigenetic modification, directly promoted chromatin gene transcription. The researchers identified 28 lactate sites on the core histones of human and mouse cells. Hypoxia and bacterial stimulation induce the production of lactate through glycolysis, and it acts as a precursor to promote histone lactylation. Using M1 macrophages exposed to bacteria as a model system, the researchers found that histone lactylation and acetylation had different time dynamics. Stimulation of mouse bone marrow-derived macrophages with lipopolysaccharide and interferon γ (LPS+IFNγ) to simulate infection with Gram-negative bacteria revealed that macrophages produced large amounts of lactate and induced histone lactylation modifications at 16–24 h of stimulation; while histone acetylation levels decreased at the same time. In the late stage of M1 macrophage polarization, the lactate modification of histones is enhanced, which induces steady-state genes involved in wound healing, including Arg1. Overall, these results suggest that the endogenous “Lactate clock” in M1 macrophages attacked by bacteria turns on gene expression to promote balance in the body (67). Histone lactylation, therefore, provides an opportunity to improve understanding of the function of lactate and its role in various pathophysiological conditions, including infection and cancer. A recent study has shown that histone lactylation accelerates tumorigenesis by activating M6A interpreter protein YTHDF2, which provides a new histone lactylation target for the treatment of ocular melanoma (73). At the same time, it also links histone modification with RNA modification, which provides a new understanding of epigenetic regulation.

### 3.2. Lactate/GPR81 signaling in cancer cells

In recent years, the importance of lactate to the survival and growth of cancer cells has been proven to be achieved in part by its ability to activate the lactate receptor (HCAR1), which is also called GPR81. GPR81 is widely distributed in human tissues. GPR81 has been identified as a lactate receptor (75). It is expressed in a variety of cell types, including adipocytes, brain cells, skeletal muscle cells, and various cancer cells (76). Xie et al. clarified the transcriptional mechanism of GPR81 expression regulation in cancer cells. Lactate induces the transcription factor Snail/STAT3 pathway and up-regulates GPR81 expression through autocrine regulation (77). A study has emphasized the importance of GPR81 in tumor growth and migration. GPR81 promotes cell proliferation and angiogenesis in a PI3K/Akt/cAMP-dependent manner in breast cancer cells. Silencing GPR81 and treating cells with PI3K inhibitors can reduce angiogenesis *in vitro*, thus inhibiting tumor growth (78). Recent work elucidates the up-regulation of PD-L1 in glucose-stimulated lung cancer cells mediated by GPR81 through lactate dehydrogenase A (LDHA). It is also proved that the activation of GPR81 reduces the level of intracellular cAMP and inhibits the activity of protein kinase A (PKA), resulting in the activation of transcriptional coactivator TAZ. The interaction between TAZ and transcription factor TEAD is the key to activating PD-L1 and inducing its expression (79). Lactate helps to protect tumor cells from being attacked by T cells, establishing the relationship between metabolic reprogramming of tumor cells and tumor evasion of the immune response. Another paper proved that immune cells are involved in the growth of GPR81-dependent tumors. The antigen is GPR81 on the surface of DC cells, and the activation of this receptor is related to the decrease of cAMP, IL-6, and IL-12, and can down-regulate the expression of MHCII on the cell surface (76). These findings suggest that lactate from tumor cells activates GPR81 in dendritic cells and blocks the expression of tumor-specific antigens to other immune cells. This paracrine mechanism complements the autocrine mechanism of PD-L1 induced by activating GPR81 in tumor cells in recent years and provides an effective means for tumor cells to evade the immune system. Therefore, blocking the GPR81 signal can promote cancer immunotherapy. GPR81 is expressed in both tumor and the surrounding immune cells at the same time, and the end result of GPR81 activation is the promotion of angiogenesis, immune evasion, and chemoresistance.

## 4. Anti-tumor metabolic therapeutic strategies targeting lactate metabolism

### 4.1. Therapeutic strategies targeting lactate metabolism

Tumor, composed of tumor cells and the TME, is currently a major scientific challenge in the field of medicine. Traditional tumor treatment methods include surgery, radiotherapy, chemotherapy, and so on, but these methods may cause serious damage to the surrounding normal tissue while killing tumor cells (80–84).

The Warburg effect is prevalent in various tumors and is characterized by a predominantly glycolytic energy metabolism in cancer cells under adequate oxygen conditions. The glycolytic product lactate can activate many essential signaling pathways in cancer cells, promoting survival, invasion, immune escape, metastasis, and angiogenesis. Combining aerobic glycolytic targeted therapy with other therapeutic approaches, such as immunotherapy and chemotherapy, is promising for cancer treatment.

Lactate oxidase (LOD) can catalyze the oxidation of lactate to pyruvate and hydrogen peroxide. The use of lactate oxidase can catalyze the change of lactate present in large amounts in tumors to H_2_O_2_, which not only dismantles the tumor immune microenvironment but H_2_O_2_ can be converted to highly toxic hydroxyl radicals (•OH) bar catalyzed by other compounds, thus killing tumor cells (85–87). The depletion of lactic acid in the tumor microenvironment by lactate oxidase can improve the tumor immunosuppressive microenvironment and effectively inhibit tumor growth.

LDH is mainly composed of different proportions of LDHA and LDHB subunits, forming five different LDH tetramer isozymes (LDH_1 − 5_). The main function of lactate dehydrogenase is to convert pyruvate to lactate and NADH to NAD^+^. The up-regulated expression of LDH can be detected in some tumor clinical samples (88–91), while the downregulation of LDH can inhibit the growth and migration of cells *in vitro* and affect the occurrence of tumors *in vivo* (92). Recently, lactate dehydrogenase-A (LDH-A) has been found to protect the dryness of cancer and recruit tumor-related macrophages to promote breast cancer progression (93). The method targeting lactate dehydrogenase is used to inhibit the production of lactate in TME.

In mouse cancer models, knockout of LDHA, LDHB, or knockout alone can inhibit tumor growth, highlighting their key role in tumor metabolism (94–96). The R- (-) enantiomeric AT-101 of gossypol acetate and its derivatives FX-11, Galloflavin, and N-hydroxyindolyl compounds have been shown to give priority to the inhibition of LDHA subtypes (21). In lymphoma and pancreatic cancer xenografts, FX-11 can reduce cellular lactate production, induce oxidative stress, and eventually lead to tumor cell apoptosis and inhibit tumor progression (97). In prostate cancer, FX-11 as a single drug can also effectively inhibit the glycolysis of tumor cells, and then inhibit the growth of tumor cells (97). It is reported that Galloflavin can bind to free LDHA and inhibit glycolysis in breast cancer cells, thus acting as an anti-tumor agent (98).

The authors identified a new LDH inhibitor, NCI-006, which overcomes many of the drawbacks of other inhibitors, is highly targeted and is expected to have an effect *in vivo* (92). N-hydroxyindole drugs have been shown to reduce the growth of pancreatic and cervical cancer cells *in vitro* (99). And when used in combination with gemcitabine, it can increase the apoptosis rate of pancreatic cancer cell lines (100).

MCTs is a proton-linked transporter responsible for transporting several monocarboxylic acid metabolites, such as pyruvate, L-lactate, and ketone bodies, across the plasma membrane with protons. MCT1/4 subtypes are dominant in tumor metabolism (101). MCTs are promising anticancer targets. The use of MCT1 inhibitors or gene knockout can interfere with lactate-fueled respiration in mitochondria (102). MCTs play an important role in the metabolic homeostasis of the tumor microenvironment. MCT1 and MCT4 are the most widely expressed MCT isoforms in cancer cells. MCT1 has a high affinity for lactate and is preferentially expressed in respiratory cancer cells that take up lactate (101). In contrast, MCT4 has a low affinity for lactate and is suitable for promoting lactate export from glycolytic cancer cells, and its expression is upregulated by hypoxia (103). The use of MCT1 inhibitors to disrupt the communication between oxidative and glycolytic cancer cells can inhibit the growth of breast cancer and promote the death of myeloma cell lines (104–106). MCTs inhibitors have been shown to reduce the invasive and migratory capacity of glioma cells (107), cervical squamous carcinoma cells (108), melanoma cells (109), triple-negative breast cancer cells (110) and pancreatic ductal adenocarcinoma cells (109). MCTs inhibitors could reduce glycolysis, lactic acid and tumor growth and allow the immune response to remain strong with increased tumor infiltration with CD8+ T and NK cells (53, 111).

As the research on TME has intensified, many kinds of synergistic therapies based on TME modulation have proliferated and achieved better therapeutic effects in mice. Recently, Zhou et al. simultaneously utilized lactate oxidase (LOD) and Fe_3_O_4_ nanoparticles (NPs) to treat tumors (86). the synergism between LOD and Fe_3_O_4_ can increase the consumption of lactate and produce more H_2_O_2_, and concurrently hydrogen peroxide (H_2_O_2_) is subsequently converted to highly toxic hydroxyl radicals (•OH) catalyzed by Fe_3_O_4_NPs *via* Fenton-like reactions to kill tumor cells. This ingenious strategy showed an obvious inhibitory effect on tumor growth and resistance to metastasis. Chen et al. effectively inhibited tumor growth and resisted tumor metastasis by using metformin (Me) and fluvastatin sodium (Flu) to interfere with lactate metabolism in tumor cells (112). On the one hand, Me alters the gluconeogenesis pathway and inhibits the tricarboxylic acid (TCA) cycle by inhibiting mitochondrial respiration (113), leading to an increase in the conversion of pyruvate to lactate. On the other hand, Flu inhibits lactate efflux, leading to intracellular acidosis, which kills tumor cells (114). Considering integrating cascaded enzymes and gene therapy, Tang et al. creatively proposed a method that can effectively inhibit tumor proliferation and angiogenesis even with the combined strategy of lactate oxidase/catalase (LOD/CAT) and vascular endothelial growth factor (VEGF) siRNA (SiVEGF) (115). The combination of lactate depletion and VEGF silencing effectively inhibited the migration of 4T1 cells *in vitro* and showed good anti-tumor and anti-metastasis properties *in vivo* (116). A recent study has shown that lactate degradation can be promoted with the assistance of lactate oxidase (LOD) cationic polyethyleneimine (PEI) and nano-coated with a certain amount of copper ion (PLNPCu). More importantly, hydrogen peroxide (H_2_O_2_), a by-product of lactate degradation, can be converted into anti-tumor ROS under the catalysis of copper ions, which mediates immunogenic cell death (ICD) (117). With the decrease of lactate in TME, the ICD process effectively promoted the anti-tumor immune response of the 4T1 tumor model (the tumor inhibition rate was 88%) (116). These strategies show a good anti-tumor effect and verify the feasibility of endogenous lactate as one of the key targets for tumor therapy. Although preclinical studies have proved that glycolysis is effective in the targeted treatment of tumors, their clinical transformation is still limited so far. The main reasons that restrict the development of glycolysis targeting therapy include metabolic heterogeneity and the damage of glycolysis targeting the immune system.

### 4.2. Therapeutic strategies targeting immune cells

Treatment of immune cells in the tumor microenvironment is also a strategy. Although there are few reports of specific treatment of immune cells, these reports have shown encouraging results in different studies. Several methods have provided promising prospects focused on targeting immune cells. INKT cells play an important role in the clearance of tumor cells (26–28). However, the tumor microenvironment affects the metabolism of INKT cells and hinders their anti-tumor functions. Fu et al. demonstrate that restoring lipid synthesis *via* activating PPARγ by using pioglitazone recovers αGC-induced IFN-γ production and significantly improves the efficacy of iNKT cell-based immunotherapy against tumors. Importantly, Pioglitazone has been already used in the treatment of type 2 diabetes, which proves its potential application in clinical antineoplastic therapy (29). This strategy of enhancing the antitumor efficacy of iNKT cell-based immunotherapy by promoting lipid biosynthesis is highly promising for clinical translation.

HIF1α can directly regulate many M2-related genes, including CD163 and ARG1 (118). Knockdown of HIF1α can greatly impair lactate-induced M2 polarization. By inhibiting the CCL17 released by TAM, they can reduce the volume of prostate cancer, reduce the invasiveness of the tumor, and the susceptibility to postoperative recurrence (37). Tumor-derived lactate induces PD-L1^+^ expression on neutrophils *via* MCT1/NF-κB/COX-2 pathway, resulting in inhibiting the efficacy of Lenvatinib. Thereby, it was believed that the COX-2 inhibitor could reduce PD- L1^+^ neutrophil and restore T cell cytotoxicity (49). Lactate can impair T cell proliferation by reducing stress independent of microenvironment acidification, which depletes the GAPDH and PGDH responses of NAD^+^ and deprives proliferative T cells of glucose-derived serine. Manipulating NAD redox metabolism may promote the differentiation and activation of T cells and open a new way to selectively promote immune regulation to enhance anti-tumor immunity (58).

GPR81 is expressed not only in cancer cells, but also in immune cells such as dendritic cells and macrophages (119). Lactate can not only affect cell proliferation, invasion, angiogenesis, and immune tolerance by activating GPR81 on tumor cells but may also promote tumor growth by activating GPR81 on non-tumor cells in the TME, as there is a functional crosstalk between tumor cells and other cells in the TME, making GPR81 a potential therapeutic target. Targeting GPR81 could inhibit the growth of cancer cells and activate the “enemy killing function” of the patient's immune system to fight the tumor. GPR81 was found closely related to tumorigenesis, development, treatment, and prognosis in clinical trials, but still, certain problems remain to be solved, such as the specific mechanism of action is not fully understood, the safety and feasibility of anti-GPR81 targeting therapy, and how to use it in clinical trials. *In vitro*, silencing GPR81 can effectively eliminate GPR81-induced angiogenesis (78). The discovery of small synthetic non-metabolite ligands for structure-based metabolite receptors is rapidly developing as many new GPCR structures are solved (120). With the maturation of experimental techniques and further research, it is believed that GPR81 will have good application prospects in the future.

## 5. Conclusions and perspectives

Lactate was neglected for a long time in the field of oncological research, but studies conducted over the past decades reveal that it plays a significant role in the development and progression of cancer by increasing angiogenesis, cancer cell migration, and metastasis. By modulating lactate in the tumor microenvironment, it was found that the GPR81 receptor inhibits host immune cells, resulting in an advanced immunological evasion by tumor cells. According to a number of research, the suppression of the anti-cancer effect of several immune cells raised by lactate deposited in the TME is transient. If extracellular lactate is depleted and the microenvironmental pH is restored, it is possible to boost the therapeutic efficacy of PD-1/PD-L1 therapeutics. Given the crucial role that lactate plays, anti-glycolytic agents such as LDHA inhibitors are capable of greatly limiting tumorigenesis. In fact, numerous other anti-tumor immune cells also rely on glycolysis to exert their functionality. Therefore, accordingly, it is assumed that anti-glycolytic inhibitors that inhibit the proliferation of tumor cells would reduce the anti-tumor immunological action of the host, rendering the treatment far less effective. Most current glycolysis inhibitors are inefficient, require high doses, and have varying degrees of damage to the immune system. Therefore, there is an urgent need to find highly effective and specific glycolytic inhibitors. Further understanding of the metabolic differences between tumor cells and immune cells is needed to identify new targeted agents that are more effective and can more appropriately modulate the immune response *in vivo*. Therapeutic regimens that target metabolism can potentially be viable and revolutionary in the fight against cancer by combining them with other therapeutic approaches such as immunotherapy and chemotherapy.

## Author contributions

ZZL, QW, XFH, and MTY designed this manuscript. ZZL, QW, XFH, MTY, ZRL, and SJZ prepared and drafted the manuscript. QW, XFH, ZRL, and SJZ prepared the figure. ZZF, QC, HJH, LL, FF, QWW, YQW, and YDT enhanced the language and analyzed the literature. AHG edited and revised manuscript. All authors contributed to the article and approved the submitted version.
